# Supplementary material for: Effect of temperatures on some biological parameters of Aphis craccivora Koch (Hemiptera: Aphididae) on lentil
Source: PeerJ. 2025 Aug 21;13:e19865. doi: 10.7717/peerj.19865 (PMC12375293; doi:10.7717/peerj.19865)
Supplement: Supplemental Information 1 [file peerj-13-19865-s001.docx]

**REFERENCES**

Aleosfoor M, and Fekrat L, (2024) Life table parameters and development of *Aphis nerii* (Hem.: Aphididae) at five different temperatures under laboratory conditions. Journal of entomological society of İran, 33(4):11-21. https://www.researchgate.net/publication/267329339.

Anonim (2023a) <https://biruni.tuik.gov.tr/medas/?kn=104&locale=tr> (Access Date: 28.02.2025)

Anonim (2023b) Turkish Journal of Agriculture and Forestry. Plant Production. <http://turktarim.gov.tr/Haber/971/dunyanin-en-kaliteli-mercimegi-turkiyede-yetisiyor->. (Access Date: 27.08.2024).

Bayhan E, Ölmez Bayhan S, Ulusoy MR and Chi H (2005a) Effect of temperature on development, mortality, fecundity, and reproduction of *Aphis rumicis* L. (Homoptera: Aphididae) on broadleaf dock (Rumex obtusifolius) and Swiss chard (Beta vulgaris vulgaris var. Cida). J Pest Sci. DOI 10.1007/s10340-005-0112-7. <https://www.researchgate.net/publication/226893402>.

Bayhan E, Ölmez Bayhan S, Ulusoy MR and Brown J.K (2005b) Effect of Temperature on the Biology of *Aphis punicae* (Passerini) (Homoptera: Aphididae) on Pomegranate. Environ. Entomol. 34(1): 22-26. <https://www.researchgate.net/publication/233642073>

Bayhan, E, Ölmez-Bayhan S, Ulusoy MR and Chi H, 2006. Effect of Temperature on Development, Mortality, Fecundity, and Reproduction of *Aphis rumicis* L. (Homoptera: Aphididae) on Broadleaf dock (*Rumex obtusifolius*) and Swiss chard (*Beta vulgaris vulgaris* var. *cida*). Journal of Pest Science, 79 (1): 57-61.

Bayındır A, Birgücü AK (2016) Effect of Temperature on Life History of *Chrysomphalus dictyospermi* (Morgan) (Hemiptera: diaspididae). DOI:https://doi.org/10.19263/REDIA-99.16.18.

Birch L.C (1948) The intrinsic rate of natural increase of an insect population. - J. Anim. Ecol., 17: 15-26.

Birgücü AK and Karsauran Y (2009) Day-Degree Models and Their Usage Possibilities in Plant Protection. ANADOLU, J. Of AARI, 12 (2), 98-117 MARA.

Campbell AB, Frazer D, Gilbert N, Gutierrez AP, Mackauer M (1974) Temperature requirements of some aphids and their parasites. J Appl Ecol 11:431–438.

Chi H (1988) Life-table analysis incorporating both sexes and variable development rates among individuals. Environmental Entomolgy, 17(1), 26-34. <https://doi.org/10.1093/ee/17.1.26>.

Chi H (2020) TWOSEX-MSChart: A computer program for the age-stage, two-sex life table analysis. Taichung, Taiwan: National Chung Hsing Universty; <http://140.120.197.173/Ecology/Dowland/Twosex-MSChart.rar>

Chi H and Liu H (1985) Two new methods for the study of insect population ecology. Bulletin of the Institute of Zoology, Academia Sinica, 24, 225-240.

Chi H, Guncan A, Kavousi A and Gholamhossein G (2022) TWOSEX-MSChart: the key tool for life table research and education. Entomologia Generalis. DOI:10.1127/entomologia/2022/1851

Dona R, Satar S (2024) Thermal effects on the biological parameters of bean *Aphis craccivora* (Hemiptera: Aphididae). DOI:https://doi.org/10.21203/rs.3.rs-4960855/v1.

Goodman D (1982) Optimal life histories, optimal notation, and the value of reproductive value. The American Naturalist 119, 803-823.

Muehlbauer FJ, Cubero JI and Summerfield RJ (1985) Lentil (*Lens culinaris* Medic.) In:R.J. Summerfield and E.H. Roberts (Eds.), Grain legume crops. Collins, 8 Grafton Street, London, UK.

Ölmez Bayhan S, Bayhan E and Ulusoy MR (2003) Effect of different temperatures on the biological parameters of *Macrosiphum rosae* (L.) (Homoptera: Aphididae). Zeitschrift für Pflanzenkrankheiten und Pflanzenschutz Journal of Plant Diseases and Protection 110 (2), 203–208, 2003, ISSN 0340-8159. Eugen Ulmer GmbH & Co., Stuttgart. https://www.researchgate.net/publication/288270063

Özgökçe M.S., Bayındır A. ve Karaca İ., (2016). Temperature-dependent development of the tomato leaf miner, Tuta absoluta (Meyrick) (Lepidoptera: Gelechiidae) on tomato plant Lycopersicon esculentum Mill. (Solanaceae). Türk. entomol. derg., 40 (1): 51-59 DOI: http://dx.doi.org/10.16970/ted.64743.

Wei-Nung Lu and Mei-Hwa Kuo, (2008). Life table and heat tolerance of *Acyrthosiphon pisum* (Hemiptera: Aphididae) in subtropical Taiwan. Entomological Science 11, 273-279. Doi:10.1111/j.1479-8298.2008.00274.x
